# Role of the subthalamic nucleus in motor control

**DOI:** 10.4103/NRR.NRR-D-25-01197

**Published:** 2025-12-30

**Authors:** Gabriel González-Escamilla, Nils Schöter, Sergiu Groppa

**Affiliations:** Department of Neurology, Saarland University, Homburg, Germany (González-Escamilla G, Schöter N, Groppa S); Department of Neurology, Saarland University Medical Center, Homburg, Germany (González-Escamilla G, Schöter N, Groppa S)

Situated below the thalamus, the subthalamic nucleus (STN) functions as an excitatory relay within the indirect pathway, influencing both the suppression of involuntary movements and fine motor execution. While the STN has traditionally been associated with motor inhibition, emerging evidence highlights its complex connectivity with cortical, striatal, pallidal, and brainstem structures, enabling precise modulation of movement intensity, timing, and coordination.

The anatomy of the STN reveals a highly organized, topographic structure, with distinct functional regions (Prasad and Wallén-Mackenzie 2024). This anatomical specificity enables the STN to integrate information across multiple domains, facilitating dynamic motor adjustments based on sensory input and cognitive demands (Herz et al., 2022). Indeed, the STN has connections to widespread brain regions, facilitating the regulation of the preparation and execution of voluntary movements (Tai et al., 2025).

Over the past few decades, accumulating evidence from both human and animal studies has highlighted the central role of the STN in motor control (Herz et al., 2022; Bange et al., 2024). Given its critical function within the basal ganglia circuits, the STN has become a prime target for deep brain stimulation (DBS) in movement disorders, particularly Parkinson’s disease. DBS of the STN effectively restores motor function by modulating abnormal brain oscillatory activity in the beta and gamma range during rest and movement, improving symptoms such as tremor and rigidity. Understanding how the STN responds to electrical modulation in DBS paradigms provides valuable insights into optimizing neurostimulation approaches for individualized patient outcomes.

This perspective presents a discussion of the anatomy of the STN, its functional role in motor at rest and during movement, and how DBS modulates its activity to alleviate motor symptoms in Parkinson’s disease. Emphasis is placed on human studies, with key findings from animal models integrated where directly relevant to understanding the underlying mechanisms. By integrating neurophysiological, computational, and clinical perspectives, this work contributes to a deeper understanding of STN-mediated motor function and therapeutic innovation in movement disorders.

**Figure 1:**
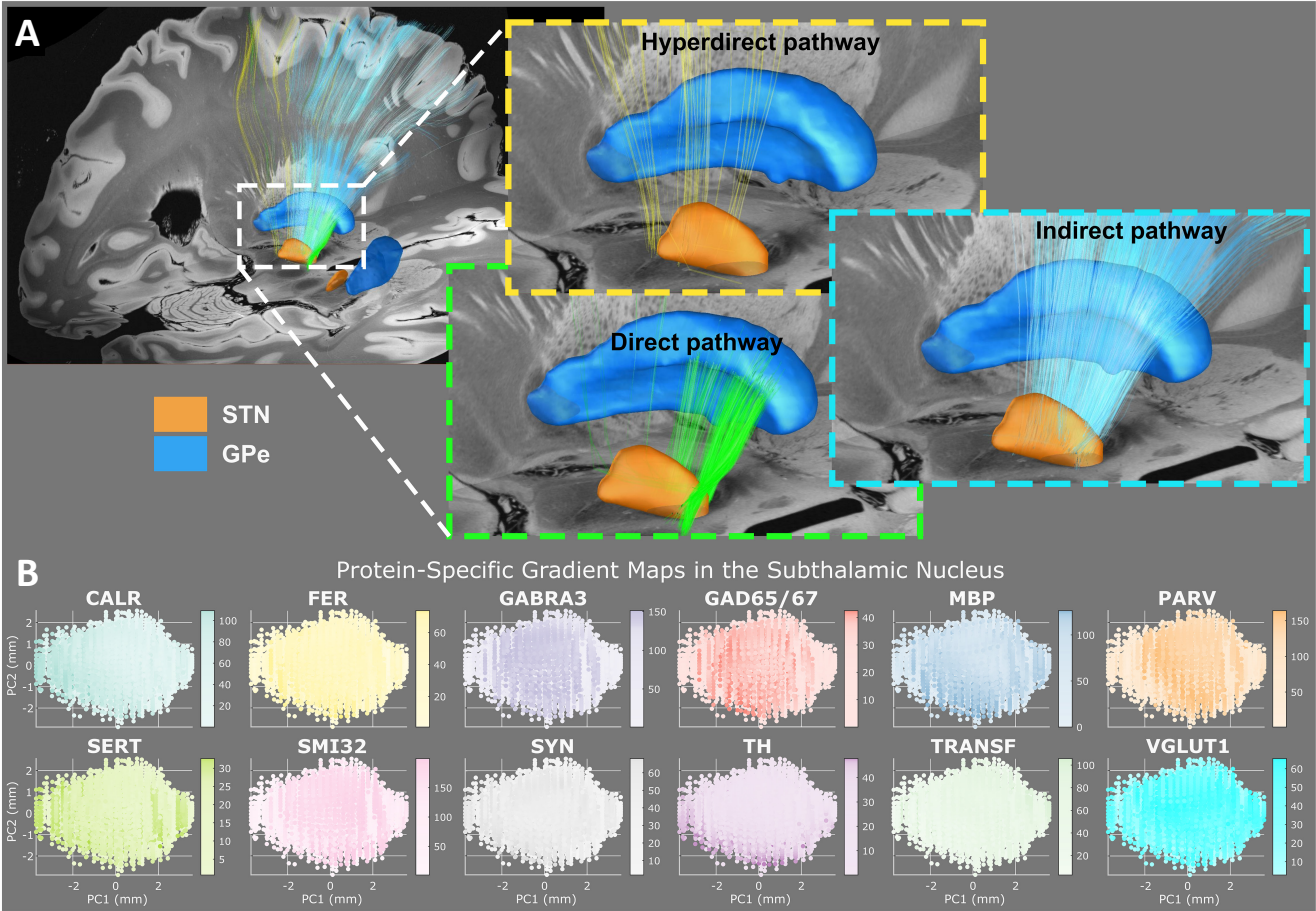
Basal ganglia circuit involving the subthalamic nucleus (STN). (A) Depiction of the basal ganglia circuits, highlighting the key motor pathways, namely the hyperdirect, the indirect, and direct pathways, involving the STN (orange). This schematic highlights the partial overlap between the motor pathways within the STN. Moreover, STN projections to prefrontal or other cortices would increase the overlap. (B) Protein marker expression of twelve proteins throughout the entire STN. For each protein marker, despite the clear local intensity differences within markers, there is a mixing spatial distribution that does not align with the proposed tripartite functional subdivisions. The depicted tracts underscore how STN connectivity spreads over the motor system, affecting overall movement control. Calretinin (CALR), ferritin (FER), GABA receptor subunit A3 (GABRA3), glutamic acid decarboxylase (GAD65/67), globus pallidus externus (GPe), myelin basic protein (MBP), parvalbumin (PARV), serotonin transporter (SERT), neurofilament H (SMI32), subthalamic nucleus (STN), synaptophysin (SYN), tyrosine hydroxylase (TH), transferrin (TRANSF), and vesicular glutamate transporter 1 (VGLUT1). The 10 × 10 × 10 grid protein data are available via https://figshare.com/s/aee5a09da245058504a9. Created using Matlab R2024b (The Mathworks, Inc).

**Anatomical and functional overview of the subthalamic nucleus:** The STN is a biconvex, lens-shaped structure situated ventral to the thalamus and adjacent to the internal capsule. While its gross appearance may seem homogeneous, neuroanatomical studies have shown a heterogeneous spatial cellular distribution that contributes to its functional diversity (Prasad and Wallén-Mackenzi, 2024). At the cellular level, the STN consists predominantly of medium to large glutamatergic neurons, interspersed with interneuronal networks that refine excitatory drive. The microstructural organization of the STN reveals a dense network of afferent fibers (**Figure 1A**) carrying input from the corticospinal tract, pallidal pathways, and serotonergic nuclei, establishing its role as a key integration hub for motor, associative, and limbic processing. The hyperdirect pathway originates in the cortex, bypasses the striatum, and directly excites STN neurons, rapidly suppressing movement through increased excitatory output to the globus pallidus internus (Gpi) and the substantia nigra pars reticulata (SNr).

The STN has been considered by some to have a strict spatial segregation into three major territories, namely, motor, associative, and limbic (i.e., the tripartite subdivision hypothesis). However, this oversimplified view, which assumes discrete STN regions are exclusively linked to specific (motor) functions, is challenged by the existence of intermingled fiber tracts (Haynes and Haber, 2013; Steiner et al., 2024) and the complex neutrotransmitter distributions (**Figure 1B**), allowing real-time integration of movement-related signals and ensuring fine-tuned control of voluntary movement. In other words, rather than being strictly spatially segregated, the STN operates through three overlapping pathways, each contributing to motor modulation in distinct ways. For instance, the hyperdirect pathway provides fast excitatory input to the STN- excites the GPi/SNr, increasing thalamic inhibition and abruptly suppressing unintended movements. This pathway is crucial for rapid stop signals, in response inhibition and motor correction. The second pathway is the direct pathway, which originates in the striatum (GABAergic neurons inhibit GPi/SNr), reduces inhibition of the thalamus, facilitating movement initiation or execution. Dopaminergic input from the SNc enhances direct pathway activity, reinforcing movement execution. The third pathway is the indirect pathway that begins in the striatum, inhibiting the globus pallidus externus (GPe). This pathway disinhibits the STN, leading to increased excitatory output to the GPi/SNr, reinforcing movement suppression.

Beyond its connectivity, its neurotransmitter profile shows that the STN is not a uniform structure with clearly delimited subregions; rather, it has a highly complex molecular architecture that may contribute to differential modulation of motor as well as non-motor processes (Haynes and Haber, 2013; Alkemade et al., 2019). The STN is primarily composed of glutamatergic projection neurons, which densely populate the structure and provide excitatory output to the GPi, GPe, and SNr. GABAergic input from the GPe is distributed throughout the STN, modulating glutamatergic excitation and preventing excessive neuronal firing, which is crucial for maintaining balanced motor control. Moreover, although the STN is not producing dopamine, it expresses dopaminergic receptors that are heterogeneously distributed throughout the nuclues (D1, D2, and D5 subtypes). Additionally, serotonergic innervation from the raphe nuclei is present throughout the STN, exerting modulatory effects on excitatory transmission and influencing behavioral regulation, impulse control, and responses to therapeutic interventions. This complex neurotransmitter interplay enables the STN to serve as a dynamic hub integrating motor, cognitive, and limbic functions within the basal ganglia circuitry.

Regarding STN function, classical basal ganglia models postulate that the STN receives both inhibitory input from the external segment of the GPe and excitatory input from the cortex. This dual modulation enables the STN to fine-tune the balance between movement initiation and suppression. Furthermore, studies using optogenetic stimulation in animal models have reinforced the idea that precise excitatory and inhibitory modulations of the STN determine the overall movement output (Friedman and Yin, 2023). Therefore, a clear understanding of the anatomical and neurochemical organization of STN is essential to correctly interpret its role in motor control.

Voluntary movement requires not only the execution of motor commands but also higher-order processes such as action selection, planning, inhibition, and error monitoring. In this direction, beyond motor-related beta (13–30 Hz) and gamma (< 30 Hz), lower-frequency oscillatory activity in the theta (4–8 Hz) and delta (< 4 Hz) range has been observed in the STN and linked to cognitive control, conflict monitoring, decision-making, and memory. For example, in PD patients, applying low-frequency STN-DBS (4 Hz) during early non-REM sleep significantly enhanced overnight declarative memory retention compared to conventional high-frequency stimulation (Herz et al., 2024). This suggests that engaging slow oscillatory dynamics in the STN can facilitate memory consolidation, likely via coordinated interactions with hippocampal-cortical networks during sleep. Such findings reflect the integrative role of STN in cognitive control, highlighting its participation in distributed circuits that support cognition.

**Subthalamic nucleus activity in motor control and rest:** Human studies have employed electrophysiological recordings to characterize STN activity at rest and movement. In patients with Parkinson’s disease, STN neurons display abnormal oscillatory patterns, particularly in the beta frequency band, which correlate with motor symptoms, especially bradykinesia and rigidity (Tai et al., 2025).

The STN plays a pivotal role in movement inhibition via the indirect pathway, preventing involuntary movements while allowing the execution of intended motor actions (Herz et al., 2022). Additionally, the hyperdirect pathway provides direct cortical excitation to the STN, also enabling rapid motor response modulation, ensuring real-time corrections and adaptability. Dysfunction of the STN, such as hyperactivity as observed in Parkinson’s disease, disrupts the balance between excitation and inhibition, leading to the characteristic motor deficits of bradykinesia, rigidity, and tremors.

**Figure 2:**
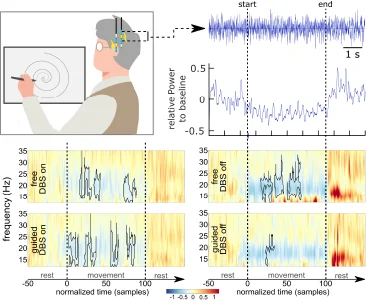
Condition- or state-dependent activation of the subthalamic nucleus. During movement, e.g., spiral drawing, local field potential signals can be registered from the implanted electrodes used for therapeutic deep neurostimulation. These signals show power changes and condition-specific patterns of burst activation (free *versus* guided movement), which are also modulated by the application of deep brain stimulation (DBS) therapy (DBS on *versus* off conditions). Created using Matlab R2024b (The Mathworks, Inc.) and modified from Bange et al. (2024).

Recently, we have characterized STN activity during both rest and movement in externalized patients (Bange et al., 2024). These data highlighted that STN neurons display abnormal burst activity patterns in the beta frequency band. Specifically, during fine motor tasks such as drawing spirals, beta bursts occur but with reduced amplitude and duration compared to resting conditions (**Figure 2**). These bursts were shown to impact movement acceleration, especially under free drawing conditions (Bange et al., 2024), thereby supporting the hypothesis of condition- or state-dependent modulation of movement by the STN.

Given the robust and consistent association between elevated resting-state beta power within the STN and motor performance in PD, these abnormal STN oscillations are considered a biomarker for dysfunctional motor control.

The findings from human recordings are further supported by animal studies using optogenetics. In controlled experiments with Parkinsonian mice, selective optogenetic inhibition of the STN resulted in an enhancement of locomotion, while excitation suppressed motor activity (Friedman and Yin, 2023). In parallel, electrophyisological studies using multi-site recordings in mice have shown that high-frequency STN stimulation selectively increased high-gamma power in motor cortical areas while reducing beta- and high-gamma coherence between STN and cortex, supporting the frequency-specific effects of STN stimulation on cortico-basal ganglia communication (Kreis et al., 2025). Although these animal models operate under non-pathological conditions, they provide valuable insights into the potential effects of modulating STN activity in humans. For instance, the reported graded, frequency-specific activation of STN projection neurons producing precise, scalable changes in movement kinematics, does not only implicates distributed output channels, contrary to discrete “territories” in the STN, but aligns with human intraoperative and chronic recording data (Steiner et al., 2024) showing that therapeutic stimulation engages circuit-level dynamics, such as evoked resonant neural activity, reflecting recruitment of extended basal ganglia-cortical loops, including hyperdirect and indirect pathways, without strict confinement to a single territory.

Notably, experiments have demonstrated that unilateral stimulation of the STN can induce rotational movements, underscoring the sensitivity of motor circuits to asymmetrical modulation. Moreover, during optogenetic excitation, behavioral phenomena such as increased self-grooming and jumping behaviors were observed—behaviors that reflect a disruption in normal coordination (Friedman and Yin, 2023). In this framework, there is a clear parallel between animal and human data, providing a mechanistic insight into how precise, parameter-dependent modulation of movement via distributed STN network engagement occurs.

The degree of modulation is not limited to active motor behavior. Even during resting periods, the STN exhibits distinct patterns of neuronal firing that differ in patients with varying stages of Parkinson’s disease (Friedman and Yin, 2023). For instance, patients with increased disease duration and symptom severity show higher STN firing rates. This suggests that disease progression is accompanied by a shift in the resting state activity of the STN, which may offer a window into potential therapeutic targets.

The interplay between STN activity during rest and voluntary movement elucidates the importance of fine-tuned neuronal oscillations in maintaining motor functionality. Elevated beta oscillations at rest are not inherently detrimental; rather, they may represent a state of optimal readiness that becomes maladaptive in the context of neurodegenerative diseases. This dual role complicates therapeutic approaches, as interventions must strike a balance between mitigating pathological oscillations and preserving essential regulatory functions.

**Role of deep brain stimulation in subthalamic nucleus function:** DBS of the STN has emerged as an effective treatment for Parkinson’s disease in patients with persistent motor fluctuations despite extensive dopaminergic medication. The rationale for employing DBS is based on its ability to modulate abnormal STN activity, thereby restoring more normalized patterns of neuronal firing, reducing pathological activity, and resulting in an overall improvement in motor symptoms (Gonzalez Escamilla et al., 2022; Bange et al., 2024).

Several electrophysiological studies have documented that STN-DBS leads to a reduction in beta oscillatory activity within the nucleus, which is associated with improvements in symptoms such as tremor, bradykinesia, and rigidity. Interestingly, DBS effectiveness is often lateralized; recordings and clinical observations suggest that the hemisphere with more pronounced beta activity is typically associated with more severe motor deficits, and targeted stimulation in this region may yield the most significant therapeutic benefit (Friedman and Yin, 2023). The modulation of STN activity via DBS thereby offers a prime example of how refined interventions can realign neuronal circuits and improve patient outcomes.

In the context of decision-making and motor planning, recent research has also explored how STN-DBS may influence cognitive dimensions of motor control. Although studies focusing on non-motor decision processes indicate that DBS can affect sensitivity to rewards and losses (Vissani et al., 2021), the primary clinical relevance remains its impact on motor performance. The convergence of cognitive and motor effects suggests that the STN is a nexus not only for motor function but also for the integration of cognitive signals that determine movement initiation and execution.

The success of DBS in modulating STN function has expanded our understanding of basal ganglia biology and underscored the importance of rhythmic neuronal activity in motor control. It also raises important questions about the optimal parameters — such as stimulation frequency, amplitude, and pulse width — that yield the best clinical outcomes. Ongoing research is aimed at deciphering these nuances and exploring the potential for tailored, patient-specific DBS protocols.

Through its hyperdirect and indirect pathway connections, the STN facilitates cognitive-motor integration, enabling the rapid adjustment of behaviour in response to changing goals or environmental demands. This dual role highlights the importance of considering interventions such as DBS in the context of both motor and cognitive network dynamics.

**Clinical implications and future directions:** The rationale for employing DBS is based on its ability to modulate abnormal STN activity, thereby restoring more physiological patterns of neuronal firing. In clinical practice, high-frequency stimulation of the STN is hypothesized to inhibit pathological activity, resulting in an overall improvement in motor symptoms. Recent studies have shown that DBS enhances acceleration and overall drawing speed during fine motor tasks. This underscores the context-dependent role of pathological beta bursts in motor control, suggesting potential for targeted therapeutic approaches such as adaptive DBS to improve motor function in patients with Parkinson’s disease (Bange et al., 2024).

The insights derived from both human electrophysiological recordings and animal model studies into STN function have several key clinical implications. First, the recognition that beta oscillations in the STN can serve as biomarkers for motor impairment has provided clinicians with an objective means to assess disease severity and tailor DBS therapy accordingly (Gonzalez-Escamilla et al., 2022; Bange et al., 2024). Moreover, the differences in lateralized STN activity suggest the potential for more refined, hemisphere-specific DBS targeting strategies that could maximize therapeutic benefits while minimizing side effects.

Furthermore, the overlap between motor and cognitive functions in the STN indicates that future therapeutic strategies may need to address both aspects concurrently. For instance, the modulation of STN activity appears to influence not only motor outputs but also decision-making processes and impulse control (Herz et al., 2022). Thus, the design of next-generation DBS devices might incorporate adaptive or closed-loop systems that adjust stimulation parameters in real time based on ongoing neural feedback. These innovations could lead to more personalized treatments that dynamically balance motor and cognitive effects.

Another important clinical consideration is the stage-dependent nature of STN dysfunction in Parkinson’s disease. Studies have revealed that the firing rate and oscillatory properties of STN neurons evolve as the disease progresses. This temporal aspect highlights the need for early biomarkers and interventions that can slow or modify disease progression. Future research that integrates long-term monitoring of STN activity with clinical outcomes may provide valuable insights into how to optimize treatments over the course of the disease.

The recognition of the involvement of STN in cognitive processes, including executive control and memory consolidation, highlights the importance of optimising DBS parameters, such as frequency, pulse width, and adaptive stimulation strategies, to maximise motor benefits and while minimizing side effects, or even induce positive effects, on cognition. Additionally, in this context, studies investigating the role of different frequency bands on both motor- and non-motor actions would clarify the exact involvement of the STN in such processes.

Finally, the challenges associated with DBS — such as the precise targeting of electrodes and the fine-tuning of stimulation parameters — underscore the need for interdisciplinary collaboration among neurosurgeons, neurologists, and biomedical engineers. Advances in imaging techniques and electrophysiological mapping will undoubtedly improve the precision of DBS implantation and lead to better patient outcomes.

**Conclusion:** The human subthalamic nucleus plays a central role in the regulation of motor control, as evidenced by both electrophysiological studies in Parkinson’s disease patients and complementary findings from animal models. The comprehensive integration of human and animal data offers a robust framework for future studies aimed at deciphering the complex interplay between neural oscillations and motor control, ultimately guiding more effective interventions for movement disorders.
